# Evaluation of age-dependent susceptibility in calves infected with two doses of *Mycobacterium avium* subspecies *paratuberculosis* using pathology and tissue culture

**DOI:** 10.1186/1297-9716-44-94

**Published:** 2013-10-07

**Authors:** Rienske AR Mortier, Herman W Barkema, Janet M Bystrom, Oscar Illanes, Karin Orsel, Robert Wolf, Gordon Atkins, Jeroen De Buck

**Affiliations:** 1 Department of Ecosystem and Public Health, University of Calgary, Hospital Drive NW, Calgary, AB, Canada; 2 Department of Biomedical Sciences, Ross University, Basseterre, Saint Kitts, West Indies

## Abstract

The longstanding assumption that calves of more than 6 months of age are more resistant to *Mycobacterium avium* subspecies *paratuberculosis* (MAP) infection has recently been challenged. In order to elucidate this, a challenge experiment was performed to evaluate age- and dose-dependent susceptibility to MAP infection in dairy calves. Fifty-six calves from MAP-negative dams were randomly allocated to 10 MAP challenge groups (5 animals per group) and a negative control group (6 calves). Calves were inoculated orally on 2 consecutive days at 5 ages: 2 weeks and 3, 6, 9 or 12 months. Within each age group 5 calves received either a high – or low – dose of 5 × 10^9^ CFU or 5 × 10^7^ CFU, respectively. All calves were euthanized at 17 months of age. Macroscopic and histological lesions were assessed and bacterial culture was done on numerous tissue samples. Within all 5 age groups, calves were successfully infected with either dose of MAP. Calves inoculated at < 6 months usually had more culture-positive tissue locations and higher histological lesion scores. Furthermore, those infected with a high dose had more severe scores for histologic and macroscopic lesions as well as more culture-positive tissue locations compared to calves infected with a low dose. In conclusion, calves to 1 year of age were susceptible to MAP infection and a high infection dose produced more severe lesions than a low dose.

## Introduction

Paratuberculosis or Johne’s disease (JD) is a chronic enteritis of ruminants caused by *Mycobacterium avium* subspecies *paratuberculosis* (MAP). Clinically affected animals have chronic, non-treatable diarrhea and wasting [1]. The major effects are reduced milk yield [2,3], premature culling and reduced slaughter value [4].

Johne’s disease in cattle has an incubation period ranging from 2 to 10 years [5]. In most cases, MAP is transmitted by the fecal-oral route [1]; in infected herds, calves are likely exposed to manure from mature cattle that shed the bacteria in their feces and to contaminated water, feed, or milk [6]. In Eastern Canada and Maine, the prevalence of MAP infection was 16.1% based on a systematic random sample of cattle at an abattoir [7].

Several studies claim cattle were infected as calves but become more resistant to the infection as they get older [8-11]. However, interpretation and comparison of the results of these studies is hindered by the low number of animals per experiment, the variety of infection doses and routes of infection used, incomplete information regarding animal housing and the variation in diagnostics used to confirm infection [12]. For this reason, proposed international guidelines were designed in order to standardize and simplify interpretation of infection experiments [13].

It has also been suggested that calves become more resistant as of 6 months of age [11,12]. As a result, current herd level control strategies focus on reducing calf exposure to all manure by recommending hygienic measures such as removing young calves from their dams to prevent transmission of MAP [6,12].

The basis for this age-dependent susceptibility has not been well documented and was recently identified as an important knowledge gap [13]. Clearly, correct understanding of age-related resistance is critical for development of effective control programs.

Because this technique can identify infection earlier than any other diagnostic test, MAP culture from gastro-intestinal tissues is considered to be the gold standard for detection of MAP infection [1] and was used to confirm infection status [14] in multiple studies.

The objective of the present study was to evaluate age- and dose-dependent susceptibility to MAP infection in dairy calves by the use of tissue culture and macroscopic and microscopic evaluation of tissues.

## Materials and methods

### Calves

Fifty-six Holstein-Friesian bull calves were purchased from 16 Alberta (Canada) dairy farms. These 16 herds were selected from 24 southern Alberta dairy herds tested to estimate the prevalence of MAP infection. Fecal and serum samples were collected from all 2nd lactation and older cows and individual milk samples were obtained simultaneously with the fecal and serum sampling through the milk recording agency CanWest DHI (Guelph, ON, Canada). To minimize the risk of including calves that had acquired intra-uterine MAP infection, calves were collected only from the 16 herds that yielded negative pooled (*n* = 5) fecal samples (decontaminated and prepared for culture according to manufacturer’s instructions; para-JEM®, TREK Diagnostic systems, OH, USA) and had a within-herd seroprevalence < 5% (IDEXX Paratuberculosis Ab Test; IDEXX Laboratories Inc, Westbrook, ME, USA).

Only calves from heifers or second parity cows and born on-farm in the presence of the research team were included in the study; contact (licking, suckling …) with the dam or environment was prevented. Additional fecal and serum samples were collected from the dam within 2 weeks after calving and tested with fecal culture and serum ELISA, respectively. Only calves from dams negative for both tests were included in the study. In addition, a precolostral serum sample was collected from each calf and tested for presence of Bovine Viral Diarrhea Virus which would indicate persistently infected calves (only calves with negative test results were used).

### Nutrition, health and husbandry

The calves were transported to the research facility and fed 6 L of gamma-irradiated colostrum (collected from fecal culture- and ELISA-negative herds) within 6 h after birth. To ensure colostrum would not contain any live MAP bacteria, this colostrum was treated with gamma irradiation with a minimum dose of 10 kGy per pail (containing 17 L of colostrum) using a Co-60 source (McMaster Nuclear Reactor, Hamilton, ON, Canada) [15]. This was followed by milk replacer and calf starter grain without antimicrobial additives and high-quality hay, as Eisenberg et al. described [16]. After weaning at 7 weeks of age, calves were fed ad libitum hay and water and supplemented with concentrates to guarantee a balanced diet.

Calves were dehorned under local anesthesia using a cauterizing iron and they were surgically castrated after administration of sedation and local anesthesia. Calves were monitored for 17 months, after which they were euthanized and necropsied.

The calves were housed in a biosecurity level 2 housing facility. This facility included 33 individual custom built housing units with waterproof liners to contain any leakage. One individual housing unit consisted of the pen containing the calf and a marked zone, in which a dedicated boot dip, boots, coveralls and gloves were provided for each individual housing unit. Calves were contained in small pens until 4 months of age after which they were individually transferred to a large animal facility – maintaining the individual housing unit set up. Personnel were trained in strict biosafety and isolation protocols to avoid transmitting MAP between calves and health status was monitored and recorded daily by clinical inspection. Animal care protocols M09083 and M09050 were approved by the Animal Care Committee of the University of Calgary.

### Study design

The 56 calves were randomly allocated to 5 age groups and 2 dose groups within each age group. Calves were inoculated with MAP at 5 ages (2 weeks, 3 months, 6 months, 9 months and 12 months). Six calves housed in the same conditions were not inoculated (negative controls). Within each of the 5 age groups containing 10 calves, 5 calves were infected with a high dose (HD) of MAP and 5 with a low dose (LD) of MAP.

The research facility allowed housing a maximum of 33 calves individually at a time. Consequently, the first 33 calves equally representing all age and dose groups, as well as 3 controls were included. The experiment was then repeated with 23 calves, including 3 control calves, also equally representing all age and dose groups.

### Inoculum

A virulent cattle type MAP strain isolated from a clinical Alberta JD case (Cow 69) was used for inoculation. This isolate has an identical BamHI, PvuII and PstI IS900 – RFLP profile to the reference strain K10 (data not shown), the strain type recommended for experimental infection trials [13]. Two doses of inoculum were used, a HD of 5 × 10^9^ CFU given on 2 consecutive days (= 5 times the recommended standard bovine challenge dose [13]) and a LD of 5 × 10^7^ CFU also given on 2 consecutive days (=10 times higher than the lowest confirmed and consistent infectious dose for young calves [14]).

Inoculum was prepared and cultured in 7H9 broth and quantified using the pelleted wet weight method, as well as qPCR as described by Eisenberg et al. [16]. Before each inoculation, one tube containing an identical aliquot of MAP cells was taken out of the -802 °C freezer and resuspended in 350 mL of 7H9 broth. The culture was incubated for exactly 7 days at 37 °C in a shaking incubator. In this period, the inoculum was tested for contamination. Right before inoculation, a 50 mL volume was prepared for the HD inoculation group and a 100-fold dilution was created for the LD inoculation group. The inoculum was placed in a syringe and expelled at the root of the tongue.

### Necropsies

Calves were euthanized at 17 months of age by intravenous injection of barbiturate (Euthanyl Forte®, DIN 00241326, Bimeda-MTC Animal Health Inc., ON, Canada) and necropsies were performed immediately. No other ruminants were allowed in the pathology room during necropsies and the necropsy room and tables were thoroughly cleaned and disinfected before and after each necropsy.

Twenty-one tissue samples were collected from each calf. For each tissue sample, a new set of disinfected instruments and clean gloves were used (to prevent cross contamination). Intestinal tissue sample locations were marked and isolated (zip ties) to prevent movement of intestinal contents. Lymph nodes (LN) were sampled before opening and sampling intestinal tissue to prevent cross contamination.

Macroscopic lesions were assessed at necropsy by a veterinary pathologist, who was blinded to the inoculation status of the calves. Macroscopic lesions were scored, based on previous studies [1,17-22], to the following categories: 0 = no macroscopic changes; 1 = one enlarged or edematous LN of the small intestine or liver; 2 = multiple enlarged and edematous mesenteric LN and/or hyperemia of the ileocaecal valve; 3 = enlarged mesenteric LN and/or mild to moderate thickening of ileal or jejunal mucosa; and 4 = enlarged mesenteric LN and severe thickening and corrugation of the ileal, jejunal and colon mucosa.

Gastro-intestinal tissue samples were collected from the duodenum, jejunum (mid and distal), ileum (proximal, mid and distal), ileocaecal valve, caecum, colon (spiral colon and transverse colon) and rectum. Lymph nodes were sampled on locations corresponding with the gastro-intestinal tract samples (except for the spiral colon, transverse colon and rectum). Additionally, the hepatic LN, tonsil, retropharyngeal LN and the superficial inguinal LN were sampled. From the 23 calves included in the second replicate, additional samples were collected (kidney, liver and spleen). Special consideration was given to evaluation of a specific subset of sampling locations: ileocaecal valve, ileocaecal LN and distal ileum. These tissue sites were considered particularly important because these were previously described as the most reliable sampling sites for MAP diagnosis [18,23,24] and these sites would provide the most reliable detection of successful infection.

From 4 specific tissue sites (ileocaecal valve, ileocaecal LN, ileal LN and distal ileum), samples were placed in a labeled cassette, immersed in 10% neutral buffered formalin solution (VWR International, Inc., Edmonton, AB, Canada) and routinely processed for histological assessment. Samples were embedded in paraffin, sectioned and stained with Hematoxylin-Eosin (HE) as well as Ziehl-Neelsen (ZN) by Prairie Diagnostic Services (Saskatoon, SK, Canada). Slides were examined by light microscopy and scored for paratuberculosis-associated histological lesions according to González et al. [25] (0 = no lesions; 1 = focal lesions; 2 = multifocal lesions; and 3 = diffuse lymphocytic, multibacillary or intermediate lesions) by an experienced veterinary pathologist who was blinded to the inoculation status of the calves.

Intestinal samples were rinsed with PBS to remove intestinal content and the mucosa was scraped off the intestinal wall using microscope slides. Fat was trimmed from the LN and these were homogenized in a Stomacher® (Stomacher® 80 Biomaster, Seward Laboratory Systems Inc., Bohemia, NY, USA) and stored at -80 °C until cultured.

### Tissue culture

From each tissue sample, 2 g was added to a sterile polyethylene stomacher bag with 5 mL of PBS and homogenized in a Stomacher®. The sample was then added to 20 mL of 0.6% hexadecylpyridinium chloride (HPC) in half strength Brain Heart Infusion (BHI). After incubation (3 h at 37 °C), tubes were centrifuged at 1700 × *g* for 20 min. Further processing was performed according to manufacturer’s recommendations (para-JEM®, TREK Diagnostic Systems). Tissue culture results were assessed in categories. Calves were assigned to the following categories: 0 = no positive tissues; 1 = 1–3 tissues positive; 2 = 4–6 tissues positive and 3 = more than 6 tissues positive.

### DNA extraction and real-time qPCR using F57

From these liquid tissue cultures, DNA was extracted as described by Forde et al. [26]. Next, real-time PCR targeting the F57 region was performed as described by Slana et al. [27] and based on Forde et al. [28]. Samples with amplification curves with a threshold cycle (Ct) below 40 were considered positive.

### Data analyses

Differences in distributions of tissue culture results, macroscopic and microscopic lesions between age and dose groups were evaluated using Chi-square and Fisher’s exact tests. For comparisons between the HD and LD groups or between specific age groups, one-sided testing was used. Agreement between the three diagnostics used was calculated with a linearly weighted kappa coefficient [29]. Analyses were performed using STATA 11.0 (StataCorp LP, College Station, TX, USA). A *P*-value ≤ 0.05 was considered significant.

## Results

### Tissue culture

Twenty-eight of the 50 (56%) inoculated calves had at least one MAP-positive tissue (Table 1). One control calf had one culture-positive tissue (duodenal LN), whereas another control calf had two culture-positive tissues (ileal LN and ileocaecal LN).

**Table 1:** Detection of MAP in calves by culture of tissues per age and dose group

Tissue culture category*	**Control**	**Low dose**					**High dose**				
		**2 weeks**	**3 months**	**6 months**	**9 months**	**12 months**	**2 weeks**	**3 months**	**6 months**	**9 months**	**12 months**
0	4	4	2	2	1	2	1	2	4	3	1
1	2	1	3	3	4	3	1	2		2	4
2							1	1	1		
3							2				

*0 = no tissue locations culture-positive; 1 = 1–3 tissue locations culture-positive; 2 = 4–6 tissue locations culture-positive; 3 = > 6 tissues culture-positive.

Positive tissue culture results were present in calves of all age and dose groups. The proportion of calves with at least one MAP-positive tissue culture, was equal (14 calves of 25; 56%) between the LD and the HD calves (*P* = 1.00). However, all 5 calves with ≥ 4 culture-positive tissues were inoculated with a HD (*P =* 0.03). The proportion of calves with at least one culture-positive tissue was similar in the 5 age groups, ranging from 40-70% (*P* = 0.82) and the proportion of tissue culture-positive calves did not decrease with increasing age at inoculation (Table 1). However, all 5 calves with ≥ 4 culture-positive tissues were inoculated at ≤ 6 months of age (*P* = 0.07).

### Tissue locations

**Figure 1:**
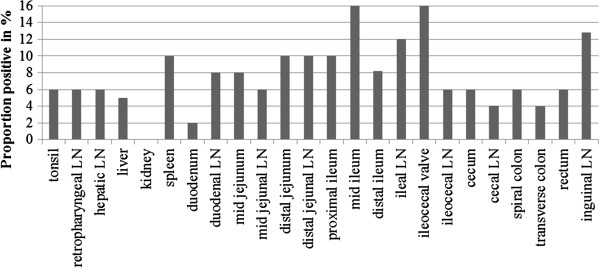
**Detection of MAP infection in calves by culture of tissues per tissue location.** The y-axis displays the proportion that one particular tissue is positive over all calves and the x-axis each tissue location sampled. LN = lymph node.

All tissue locations were MAP culture-positive in at least one calf, except for the kidney, although no location was MAP culture-positive in all inoculated calves (Figure 1). The proportion of positive tissue sites ranged from 0% (kidney) to 16% (mid ileum and ileocaecal valve); most sampling sites in this trial were culture-positive in < 10% of the MAP- inoculated calves (Figure 1).

**Figure 2:**
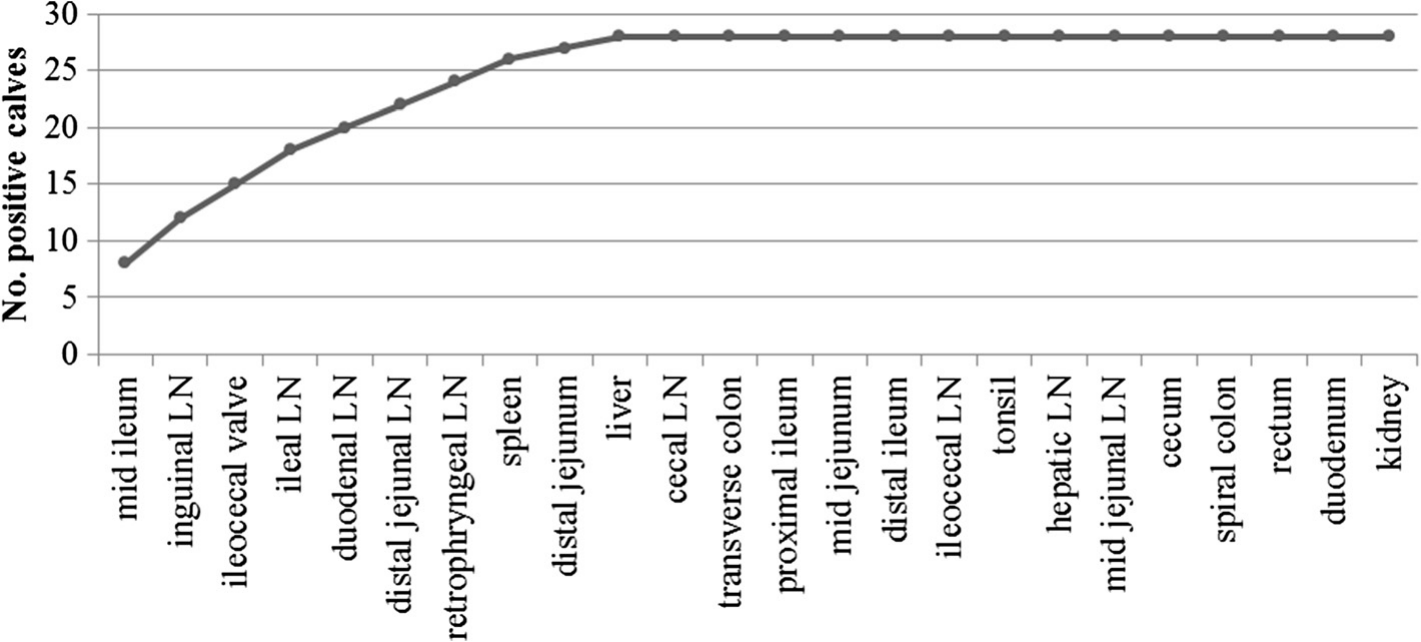
**Number of calves detected as MAP culture-positive per additional tissue location sampled.** Tissue locations are ordered on the x-axis starting with the location that detected the most MAP-infected calves (y-axis) to the tissue location that yielded the least number of calves positive. LN = lymph node.

A minimum of 10 tissue locations was necessary to identify all culture-positive calves. Any extra samples did not increase the number of calves detected (Figure 2). Using the subset of tissue locations most frequently used to diagnose MAP infection, ileocaecal valve, ileocaecal LN and distal ileum, 8 (31%) of the 26 calves with at least one MAP-positive tissue culture were detected. All of these 8 calves were MAP culture-positive for the ileocaecal valve and from these, 4 also had a positive culture for the distal ileum, whereas 3 had a positive culture for the ileocaecal LN.

By the end of the trial, two HD calves inoculated at 2 weeks of age had clinical symptoms of JD; these calves both had 18 of 24 culture-positive tissue locations.

### Macroscopic lesions

Macroscopic lesions were present in calves in all age and dose groups (Table 2). Thirty-one (62%) of 50 inoculated calves had macroscopic lesions, whereas no gross lesions were detected in the control calves (*P* = 0.005). Of the 25 calves inoculated with a HD, 16 (64%) of 25 had a macroscopic lesion score > 2 compared to 9 (36%) of the 25 LD calves (*P* = 0.04). The proportion of calves with macroscopic lesions differed among age groups (Table 2; *P* = 0.03). However, more calves inoculated at 12 months of age had macroscopic lesions compared to 9-months inoculated calves (*P* = 0.03).

**Table 2:** Macroscopic lesions of MAP infection in calves (according to age and dose groups)

Macroscopic lesions*	**Control**	**Low dose**					**High dose**				
		**2 weeks**	**3 months**	**6 months**	**9 months**	**12 months**	**2 weeks**	**3 months**	**6 months**	**9 months**	**12 months**
0	6	1	1	3	5	2		1	2	3	1
1		1	1	1					1		
2			1				1				
3		3	2	1		3	2	4	2	2	4
4							2				

*0 = no macroscopic changes; 1 = one enlarged or edematous lymph node of the small intestine or liver; 2 = multiple enlarged and edematous mesenteric lymph nodes and/or hyperemia of the ileocaecal valve; 3 = enlarged mesenteric lymph node(s) and/or mild to moderate thickening of ileal or jejunal mucosa; 4 = enlarged mesenteric lymph node(s) and severe thickening and corrugation of the ileal, jejunal and colon mucosa.

Both of the 2 HD calves inoculated at 2 weeks of age that had clinical symptoms of JD had severe (category 4) macroscopic lesions.

### Histology

Histological lesions in samples of the ileocaecal valve, ileocaecal LN, ileal LN and distal ileum were present in calves in all age and dose groups (Table 3). Forty-two (84%) of 50 MAP-inoculated calves had histological lesions which, with the exception of 2 animals, were classified as mild and focal within affected tissues. Subtle and focal lesions (occasional tiny clusters of epithelioid ZN-negative macrophages) were also detected in 4 of 6 control calves. Twenty (80%) calves in the LD and 22 (88%) calves in the HD groups had lesions (*P =* 0.35).

**Table 3:** Number of calves per histology category in each age and dose group

		**Low dose**					**High dose**				
Histology*	**Control**	**2 weeks**	**3 months**	**6 months**	**9 months**	**12 months**	**2 weeks**	**3 months**	**6 months**	**9 months**	**12 months**
0	2	1	1		3					2	1
1	4	3	4	5	2	5	2	3	5	3	4
2		1					1	2			
3							2				

*0 = no lesions; 1 = focal lesions; 2 = multifocal lesions; 3 = diffuse lymphocytic, multibacillary or intermediate lesions.

Six (12%) of 50 inoculated calves had extensive histological lesions (scores 2 or 3; Table 3). These extensive histological lesions were only present in calves in the 2-weeks group and 3-months group (*P* = 0.002), but not in the other age groups. Also, most calves (5 of 6) with histology score 2 or 3 were inoculated with a HD. The 6 calves with extensive histological lesions were also the only calves that were ZN-positive. The 2 calves in the 3-months HD group had lesions in 3 of 4 tissues; both were positive in the ileal LN with the ZN stain. The two non-clinical 2-weeks calves had lesions in 3 of 4 tissues; one had a ZN-positive ileocaecal valve, and the second calf was ZN-positive in 3 of 4 tissues.

Both HD calves inoculated at 2 weeks of age that had clinical symptoms of JD had severe histological lesions in all 4 tissues and were strongly ZN-positive in all 4 tissues.

### Tissue culture, macroscopy and histology combined

Forty-three (86%) of 50 MAP-inoculated calves were positive on at least one diagnostic test, represented in all age and dose groups (Table 4), whereas 2 control calves were also positive on one of the 3 tests (with a histology score ≥ 2 considered positive). Twenty-two (44%) calves in the LD and 21 (42%) in the HD groups were identified with either tissue culture, histology (score > 2) or macroscopy (*P* = 1.00). All 5 age groups had calves positive on at least one of 3 tests, ranging from 70 to 100% (*P* = 0.12).

**Table 4:** Number of calves positive on either MAP tissue culture, histology or macroscopy in each age and dose group

Positive*	**Control**	**Low dose**					**High dose**				
		**2 weeks**	**3 months**	**6 months**	**9 months**	**12 months**	**2 weeks**	**3 months**	**6 months**	**9 months**	**12 months**
0	4	1		1	1				2	2	
1	2	4	5	4	4	5	5	5	3	3	5

*Tissue culture or histology (score >2) or macroscopic score.

### Association between tissue culture, macroscopic lesions and histology

The highest agreement was between histopathology findings and tissue culture (80%) (Table 5), but kappa values were all <0.2 indicating slight agreement between the 3 diagnostic methods.

**Table 5:** Linearly weighted kappa coefficients between histology, macroscopic lesions and MAP tissue culture

	**Agreement**	**Kappa**	**95% CI of kappa**
Macroscopy – tissue culture	66%	0.08	-0.06 - 0.14
Macroscopy – histology	70%	0.17	0.13 - 0.24
Histology – tissue culture	80%	0.19	-0.11 - 0.27

CI = Confidence interval.

## Discussion

In all age and dose groups at least one calf had a positive sample for tissue culture, macroscopic lesions or histology. Calves inoculated at a younger age had higher scores for histology and tissue culture, while calves inoculated at an older age were more severely affected macroscopically. Also, only a slight agreement was present between the three diagnostic methods used. Furthermore, a low proportion of tissue sites was culture-positive and multiple tissues were needed to identify a calf as infected.

Based on a previous study [12], the proportion of successfully infected calves was expected to be 75% in calves < 6 months of age, 50% of calves 6 to 12 months of age, and 20% of cattle > 12 months of age. Based on that study, it would only be possible to successfully infect older calves with a HD [12]. In the present study, in all age groups, inoculated from 2 weeks to 12 months, a high proportion of animals became infected, even with a LD. Similarly, when 1 to 2-year-old cattle grazed on pasture previously grazed by MAP-infected cattle, these yearlings also became MAP-infected [30]. Susceptibility of adult animals might have been missed in a previous trial, as adults were not included due to their expected resistance to MAP infection [31].

Calves inoculated at 6 months or younger had more culture-positive tissue locations and slightly more prominent histological lesions. The follow-up period of calves inoculated at 12 months of age was 5 months, whereas calves inoculated at 2 weeks were necropsied 16.5 months after inoculation. The observed difference in severity could, therefore, also be due to differences among groups in follow-up. As expected, calves infected with a HD had higher histological and macroscopic lesion scores, as well as more culture-positive tissue locations compared to calves infected with a LD.

No age-dependent susceptibility to MAP infection was detected in a small ruminant study [32]. The resulting guidelines for small ruminant control programs pragmatically suggest to “keep in mind that while young animals are the most susceptible to infection, small ruminants can be infected as adults and may succumb to JD at any age”. This is in agreement with findings in deer [33], and it can be concluded that older ruminants are still susceptible to MAP infection.

In the absence of an absolute age-related resistance, other mechanisms should be considered that explain the observed variability in individual host response to MAP infection. In cattle, genetic susceptibility markers have been identified [34-38]. Consequently, marker-assisted breeding might be implemented in JD prevention and control strategies [38]. Genetic susceptibility traits have also been found in deer; some breeds of red deer vary in resistance/susceptibility [39]. Based on these previous findings, we speculated that the genetic variation related to the regulation of the cellular immune response could be responsible for observed differences in host responses.

Remarkably, calves at all ages were successfully infected with either a HD or a LD of MAP. However, calves given a HD of MAP had more culture-positive tissue locations as well as slightly more prominent macroscopic and microscopic lesions. Furthermore, it was noteworthy that the two calves with the highest number of positive tissue locations and the highest scoring histological and macroscopical lesions were inoculated with a HD. This dose dependency, also reported in sheep [40], emphasizes the importance of keeping the infection pressure low-to minimize transmission.

In this study, tissue culture, histology and gross lesions results did not always match on an individual animal level. However, there was a positive association between tissue culture and the relative severity of histological findings consistent with a previous study [41]. Also, deer as well as cattle with macroscopic lesions generally have a higher likelihood of culture-positive tissue, but MAP infection could still be detected in animals with macroscopically normal tissues [41-43], as was also the case in the present study. If tissue culture is combined with histopathology, detection of MAP infection is improved [41]. Furthermore, tissue culture was a far superior diagnostic tool to the use of ZN acid fast stain, as previously described [44]. However, in contrast to Brady et al. [21], MAP was not detected in all grossly affected tissues in this study, nor was MAP detected in all tissues with histological lesions. That MAP was absent in gross and histological lesions could have been due to a lesion not caused by MAP, or too few MAP were present in that site and hence not successfully cultured, or cure could have occurred.

Dissemination of MAP is usually not expected until the clinical stage [45]; however MAP was distributed in clinically normal animals [21]. Consequently, it was suggested that restricting tissue sampling to the ileum and ileocaecal LN would decrease detection of many infected animals [46] and routine culture should be extended from the GI tract to other LN and tissues [47]. Furthermore, Buergelt et al. [18] and Condron et al. [48] both suggested that MAP was disseminated in subclinical infections [46] and we concluded that dissemination of MAP soon after infection (during the subclinical stage) might be much more widespread than commonly believed.

Although tissue culture is considered the gold standard and capable of detecting a MAP-infected animal before other diagnostic tests [1], only half (56%) of challenged calves were tissue culture-positive in this study. There are other reports of low detection rates of MAP in infected tissues [46], especially when the number of tissue samples is limited. This low detection rate of infected animals may lead to underestimation of the true prevalence of MAP infection as well as underestimation of test agreement when using tissue culture as a gold standard. Furthermore, restricting tissue samples to ileum and ileocaecal LN will also underestimate the number of infected animals not yet expressing clinical signs [46,47].

Statistical analyses could have been more meaningful if a logistic regression with interaction between dose and age could have been performed for each diagnostic test used. However, even though this was a study of considerable size, the number of calves in each category was too low to make this analysis possible. Another asset that would have added to this study is quantification of MAP-bacteria in the tissues by means of the time-to-positive signals. However, this was not done because the interpretation of the pressure curves by the liquid culture system is optimized for fecal culture and failed to perform well on cultured tissues.

Two of 6 non-inoculated calves had positive tissue culture results and 4 control calves had focal histological lesions. Perhaps presence of one focal lesion was a non-specific inflammatory response not due to MAP infection, and may correspond to a small clusters of what are known as “garbage” macrophages [25], especially because these lesions were found in the paracortex of the LN only. In one control calf, MAP was detected from the 2 LN that contained focal lesions microscopically, whereas another calf with a focal lesion in the ileocaecal LN was shedding at 3 time points during the trial as well, suggesting a true infection. A false-positive PCR result is unlikely as the MAP-specific F57 target was used [27]. Even though all calves in this study were housed individually, and strict biosecurity measures were applied to avoid cross-contamination, perhaps MAP was transferred from the infected calves to the control calves. Recently, dust has been suggested as a means of transmission for MAP [49-52]. Although the hay fed to the calves may have been contaminated with MAP, this was considered unlikely, as the hay was fed harvested from fields not grazed by cattle for several years. In utero infection is a possibility [53], despite considerable efforts to use calves with the lowest probability of an intrauterine infection. Although every effort was done to prevent contamination from calf to calf, it is also possible that these calves acquired MAP infection during the experiment. Due to insufficient DNA present after F57 PCR, it was unfortunately not possible to genotype the isolates from control calves and gain knowledge on the source of infection. If more stringent criteria were used to determine infection status using tissue culture and histology based on the results from the control animals, only calves infected at or < 6 months of age with a high dose were successfully infected with MAP (Tables 1 and 3). However, the shorter follow-up period of the 9 and 12 months infection group might lead to an underestimation of infection in these animals.

Based on the results of the present study, we conclude that JD prevention and control programs should emphasize lowering MAP infection pressure, as it was proven that a lower infection dose resulted in less severe lesions. Furthermore, since cattle up to at least 1 year of age are susceptible to MAP infection, prevention of infection should include calves of all ages.

## Abbreviations

BHI: Brain heart h infusion; HD: High dose; HPC: Hexadecylpyridinium chloride; HE: Hematoxylin-Eosin; JD: Johne’s disease; LD: Low dose; LN: Lymph node; MAP: *Mycobacterium avium* subspecies *paratuberculosis*; ZN: Ziehl-Neelsen.

## Competing interests

The authors declare that they have no competing interests.

## Authors’ contributions

Designed and conducted the experiment: RM, HWB, KO, JDB. Developed and took care of inoculum preparation and the inoculation procedure, collected and analysed the samples, performed necropsies: RM. Gross lesion assessment: JB. Histology assessment: OI. Animal management, health and welfare: RM, KO, RW, GA, JDB. Analysis of data: RM, HB. Drafted the manuscript: RM, HWB, JDB. All authors read and approved the final manuscript.

## References

[B1] Tiwari A VanLeeuwen JA McKenna SL Keefe GP Barkema HW Johne’s disease in Canada Part I: clinical symptoms, pathophysiology, diagnosis, and prevalence in dairy herds Can Vet J 2006 44 874 882 17017652 PMC1555680

[B2] Benedictus G Dijkhuizen AA Stelwagen J Economic losses due to paratuberculosis in dairy cattle Vet Rec 1987 44 142 146 10.1136/vr.121.7.142 3660545

[B3] Kudahl A Nielsen SS Sorensen JT Relationship between antibodies against *Mycobacterium avium* subsp. *paratuberculosis* in milk and shape of lactation curves Prev Vet Med 2004 44 119 134 10.1016/j.prevetmed.2003.11.008 15156998

[B4] Chi J VanLeeuwen JA Weersink A Keefe GP Direct production losses and treatment costs from bovine viral diarrhoea virus, bovine leukosis virus, *Mycobacterium avium* subspecies *paratuberculosis*, and *Neospora caninum* Prev Vet Med 2002 44 137 153 10.1016/S0167-5877(02)00094-6 12350317

[B5] Whitlock RH Buergelt C Preclinical and clinical manifestations of paratuberculosis (including pathology) Vet Clin North Am Food Anim Pract 1996 44 345 356 8828109 10.1016/s0749-0720(15)30410-2

[B6] McKenna SL Keefe GP Tiwari A VanLeeuwen J Barkema HW Johne’s disease in Canada part II: disease impacts, risk factors, and control programs for dairy producers Can Vet J 2006 44 1089 1099 17147140 PMC1624920

[B7] McKenna SL Keefe GP Barkema HW McClure J Vanleeuwen JA Hanna P Sockett DC Cow-level prevalence of paratuberculosis in culled dairy cows in Atlantic Canada and Maine J Dairy Sci 2004 44 3770 3777 10.3168/jds.S0022-0302(04)73515-8 15483160

[B8] Hagan WA Age as a factor in susceptibility to Johne’s Disease Cornell Vet 1938 44 34 40

[B9] Larsen AB Merkal RS Cutlip RC Age of cattle as related to resistance to infection with *Mycobacterium paratuberculosis* Am J Vet Res 1975 44 255 257 1115424

[B10] Rankin JD The experimental infection of cattle with *Mycobacterium johnei*. IV. Adult cattle maintained in an infectious environment J Comp Pathol 1962 44 113 117 14490277 10.1016/s0368-1742(62)80013-7

[B11] Taylor AW Experimental Johne’s Disease in cattle J Comp Pathol Ther 1953 44 355 373 10.1016/s0368-1742(53)80037-8 13109041

[B12] Windsor PA Whittington RJ Evidence for age susceptibility of cattle to Johne’s disease Vet J 2010 44 37 44 10.1016/j.tvjl.2009.01.007 19246220

[B13] Hines ME 2nd Stabel JR Sweeney RW Griffin F Talaat AM Bakker D Benedictus G Davis WC de Lisle GW Gardner IA Juste RA Kapur V Koets A McNair J Pruitt G Whitlock RH Experimental challenge models for Johne’s disease: a review and proposed international guidelines Vet Microbiol 2007 44 197 222 10.1016/j.vetmic.2007.03.009 17467201

[B14] Sweeney RW Uzonna J Whitlock RH Habecker PL Chilton P Scott P Tissue predilection sites and effect of dose on *Mycobacterium avium* subs. *paratuberculosis* organism recovery in a short-term bovine experimental oral infection model Res Vet Sci 2006 44 253 259 10.1016/j.rvsc.2005.07.007 16165171

[B15] Garin-Bastuji B Perrin B Thorel M-F Martel JL Evaluation of γ-rays irradiation of cows’ colostrum for *Brucella abortus*, *Escherichia coli* K99, *Salmonella dublin* and *Mycobacterium paratuberculosis* decontamination Lett Appl Microbiol 1990 44 163 166 10.1111/j.1472-765X.1990.tb00150.x

[B16] Eisenberg SW Koets AP Nielen M Heederik D Mortier R De Buck J Orsel K Intestinal infection following aerosol challenge of calves with *Mycobacterium avium* subspecies *paratuberculosis* Vet Res 2011 44 117 10.1186/1297-9716-42-117 22136728 PMC3245454

[B17] Clarke CJ The pathology and pathogenesis of paratuberculosis in ruminants and other species J Comp Pathol 1997 44 217 261 10.1016/S0021-9975(97)80001-1 9147244

[B18] Buergelt CD Hall C McEntee K Duncan JR Pathological evaluation of paratuberculosis in naturally infected cattle Vet Pathol 1978 44 196 207 10.1177/030098587801500206 664186

[B19] Payne JM Rankin JD A comparison of the pathogenesis of experimental Johne’s disease in calves and cows Res Vet Sci 1961 44 175 179

[B20] Payne JM Rankin JD The pathogenesis of experimental Johne’s disease in calves Res Vet Sci 1961 44 167 174

[B21] Brady C O’Grady D O’Meara F Egan J Bassett H Relationships between clinical signs, pathological changes and tissue distribution of *Mycobacterium avium* subspecies *paratuberculosis* in 21 cows from herds affected by Johne’s disease Vet Rec 2008 44 147 152 10.1136/vr.162.5.147 18245746

[B22] Okuni JB Reinacher M Loukopoulos P Ojok L Prevalence and spectrum of Johne’s disease lesions in cattle slaughtered at two abattoirs in Kampala, Uganda Trop Anim Health Prod 2012 44 1197 1202 23274696 10.1007/s11250-012-0346-3

[B23] Benedictus G Bosma J Paratuberculosis: a surgical method of diagnosis in practice Vet Q 1985 44 217 221 10.1080/01652176.1985.9693985 3901498

[B24] Julian RJ A short review and some observations on Johne’s disease with recommendations for control Can Vet J 1975 44 33 43 1093661 PMC1696802

[B25] González J Geijo MV García-Pariente C Verna A Corpa JM Reyes LE Ferreras MC Juste RA García Marín JF Pérez V Histopathological classification of lesions associated with natural paratuberculosis infection in cattle J Comp Pathol 2005 44 184 196 10.1016/j.jcpa.2005.04.007 16045917

[B26] Forde T Kutz S De Buck J Warren A Ruckstuhl K Pybus M Orsel K Occurrence, diagnosis, and strain typing of *Mycobacterium avium* subspecies *paratuberculosis* infection in rocky mountain bighorn sheep (*Ovis canadensis canadensis*) in southwestern Alberta J Wildl Dis 2012 44 1 11 10.7589/0090-3558-48.1.1 22247368

[B27] Slana I Kralik P Kralova A Pavlik I On-farm spread of *Mycobacterium avium* subsp. *paratuberculosis* in raw milk studied by IS900 and F57 competitive real time quantitative PCR and culture examination Int J Food Microbiol 2008 44 250 257 10.1016/j.ijfoodmicro.2008.08.013 18824269

[B28] Forde T De Buck J Elkin B Kutz S van der Meer F Orsel K *Mycobacterium avium* subspecies *paratuberculosis* in wood bison: contrasting results of culture-dependent and molecular analyses Appl Environ Microbiol 2013 44 4448 4454 10.1128/AEM.00995-13 23686265 PMC3697503

[B29] Brenner H Kliebsch U Dependence of weighted kappa coefficients on the number of categories Epidemiology 1996 44 199 202 10.1097/00001648-199603000-00016 8834562

[B30] Fecteau ME Whitlock RH Buergelt CD Sweeney RW Exposure of young dairy cattle to *Mycobacterium avium* subsp. *paratuberculosis* (MAP) through intensive grazing of contaminated pastures in a herd positive for Johne’s disease Can Vet J 2010 44 198 200 20436867 PMC2808288

[B31] Mitchell RM Medley GF Collins MT Schukken YH A meta-analysis of the effect of dose and age at exposure on shedding of *Mycobacterium avium* subspecies *paratuberculosis* (MAP) in experimentally infected calves and cows Epidemiol Infect 2012 44 231 246 10.1017/S0950268811000689 21524342

[B32] Robbe-Austerman S Control of paratuberculosis in small ruminants Vet Clin North Am Food Anim Pract 2011 44 609 620 vi 10.1016/j.cvfa.2011.07.007 22023839

[B33] Mackintosh CG Clark RG Thompson B Tolentino B Griffin JF de Lisle GW Age susceptibility of red deer (*Cervus elaphus*) to paratuberculosis Vet Microbiol 2010 44 255 261 10.1016/j.vetmic.2009.11.014 20005645

[B34] Koets AP Adugna G Janss LL van Weering HJ Kalis CH Wentink GH Rutten VP Schukken YH Genetic variation of susceptibility to *Mycobacterium avium* subsp. *paratuberculosis* infection in dairy cattle J Dairy Sci 2000 44 2702 2708 10.3168/jds.S0022-0302(00)75164-2 11104291

[B35] Kirkpatrick BW Shi X Shook GE Collins MT Whole-Genome association analysis of susceptibility to paratuberculosis in holstein cattle Anim Genet 2010 44 149 160 10.1111/j.1365-2052.2010.02097.x 20618184

[B36] Gonda MG Kirkpatrick BW Shook GE Collins MT Identification of a QTL on BTA20 affecting susceptibility to *Mycobacterium avium* ssp. *paratuberculosis* infection in US Holsteins Anim Genet 2007 44 389 396 10.1111/j.1365-2052.2007.01627.x 17617211

[B37] Ruiz-Larranaga O Garrido JM Manzano C Iriondo M Molina E Gil A Koets AP Rutten VP Juste RA Estonba A Identification of single nucleotide polymorphisms in the bovine solute carrier family 11 member 1 (SLC11A1) gene and their association with infection by *Mycobacterium avium* subspecies *paratuberculosis* J Dairy Sci 2010 44 1713 1721 10.3168/jds.2009-2438 20338449

[B38] Koets A Santema W Mertens H Oostenrijk D Keestra M Overdijk M Labouriau R Franken P Frijters A Nielen M Rutten V Susceptibility to paratuberculosis infection in cattle is associated with single nucleotide polymorphisms in Toll-like receptor 2 which modulate immune responses against *Mycobacterium avium* subspecies *paratuberculosis* Prev Vet Med 2010 44 305 315 10.1016/j.prevetmed.2009.11.008 20005587

[B39] Dobson B Liggett S O’Brien R Griffin JF Innate immune markers that distinguish red deer (*Cervus elaphus*) selected for resistant or susceptible genotypes for Johne’s disease Vet Res 2013 44 5 10.1186/1297-9716-44-5 23347398 PMC3574005

[B40] Delgado L Juste RA Munoz M Morales S Benavides J Ferreras MC Marin JF Perez V Differences in the peripheral immune response between lambs and adult ewes experimentally infected with *Mycobacterium avium* subspecies *paratuberculosis* Vet Immunol Immunopathol 2012 44 23 31 10.1016/j.vetimm.2011.10.005 22070826

[B41] Elze J Liebler-Tenorio E Ziller M Kohler H Comparison of prevalence estimation of *Mycobacterium avium* subsp. *paratuberculosis* infection by sampling slaughtered cattle with macroscopic lesions vs. systematic sampling Epidemiol Infect 2013 44 1536 1544 10.1017/S0950268812002452 23148821 PMC9151621

[B42] Hunnam J Wilson P Heuer C Stringer L Clark R Mackintosh C Association between *Mycobacterium avium* subspecies *paratuberculosis* and lymph node size in New Zealand farmed deer (*Cervus elaphus*) N Z Vet J 2013 44 133 140 10.1080/00480169.2012.755886 23442016

[B43] Stringer L Wilson P Heuer C Hunnam J Verdugo C Mackintosh C Prevalence of *Mycobacterium avium* subsp. *paratuberculosis* in farmed red deer (*Cervus elaphus*) with grossly normal mesenteric lymph nodes N Z Vet J 2013 44 147 152 10.1080/00480169.2012.755888 23441922

[B44] Martinson SA Hanna PE Ikede BO Lewis JP Miller LM Keefe GP McKenna SL Comparison of bacterial culture, histopathology, and immunohistochemistry for the diagnosis of Johne’s disease in culled dairy cows J Vet Diagn Invest 2008 44 51 57 10.1177/104063870802000109 18182508

[B45] Chiodini RJ Immunology: resistance to paratuberculosis Vet Clin North Am Food Anim Pract 1996 44 313 343 8828108 10.1016/s0749-0720(15)30409-6

[B46] Whitlock RH Rosenberger AE Sweeney RW Spencer PA Chiodini RJ MEHII, Collins MT Distribution of *M. paratuberculosis* in tissues of cattle from herds infected with Johne’s disease Fifth International Colloquium on Paratuberculosis; September 29 - October 4 Madison, Wisconsin, USA 1996 168 173

[B47] Pavlik I Matlova L Bartl J Svastova P Dvorska L Whitlock R Parallel faecal and organ *Mycobacterium avium* subsp. *paratuberculosis* culture of different productivity types of cattle Vet Microbiol 2000 44 309 324 10.1016/S0378-1135(00)00316-3 11118716

[B48] Condron R Schroen C Black C Ridge S Hope A Chiodini RJ, Collins MT, Bassey EOE Histological confirmation of subclinical infection with *M*. *paratuberculosis* in cattle Proceedings of the Fourth International Colloquium on Paratuberculosis 1994 37 40

[B49] Eisenberg SW Nielen M Hoeboer J Rutten V Heederik D Koets AP Environmental contamination with *Mycobacterium avium* subspecies *paratuberculosis* within and around a dairy barn under experimental conditions J Dairy Sci 2012 44 6477 6482 10.3168/jds.2012-5548 22939786

[B50] Eisenberg S Nielen M Hoeboer J Bouman M Heederik D Koets A *Mycobacterium avium* subspecies *paratuberculosis* in bioaerosols after depopulation and cleaning of two cattle barns Vet Rec 2011 44 587 10.1136/vr.d1091 21610001

[B51] Eisenberg SW Koets AP Hoeboer J Bouman M Heederik D Nielen M Presence of *Mycobacterium avium* subsp. *paratuberculosis* in environmental samples collected on commercial Dutch dairy farms Appl Environ Microbiol 2010 44 6310 6312 10.1128/AEM.00998-10 20656861 PMC2937508

[B52] Eisenberg SW Nielen M Santema W Houwers DJ Heederik D Koets AP Detection of spatial and temporal spread of *Mycobacterium avium* subsp. *paratuberculosis* in the environment of a cattle farm through bio-aerosols Vet Microbiol 2010 44 284 292 10.1016/j.vetmic.2009.11.033 20036081

[B53] Whittington RJ Windsor PA In utero infection of cattle with *Mycobacterium avium* subsp. *paratuberculosis*: a critical review and meta-analysis Vet J 2009 44 60 69 10.1016/j.tvjl.2007.08.023 17928247

